# Healthcare delivery to patients from culturally and linguistically diverse backgrounds in emergency care: a scoping review protocol

**DOI:** 10.1186/s13643-024-02579-0

**Published:** 2024-07-12

**Authors:** Ya-Ling Huang, Sarah Thorning, Chun-Chih Lin, Robert Lee, Elizabeth Elder, Julia Crilly

**Affiliations:** 1 School of Nursing and Midwifery, University of Southern Queensland, Ipswich, QLD 4305 Australia; 2 Department of Emergency Medicine, Gold Coast Hospital and Health Service, Gold Coast, QLD Australia; 3 Faculty of Health (Nursing), Southern Cross University, Gold Coast, QLD Australia; 4 School of Nursing and Midwifery, Griffith University, Gold Coast, QLD Australia; 5 Education and Research Unit, Central Queensland Hospital and Health Service, Rockhampton, QLD Australia; 6 Department of Nursing, Chang Gung University of Science and Technology, ChiaYi, Taiwan; 7 New Taipei Municipal TuCheng Hospital, Chang Gung Medical Foundation, New Taipei City, Taiwan; 8 Consumer Advisory Group, Gold Coast Hospital and Health Service, Gold Coast, QLD Australia; 9 Menzies Health Institute Queensland, Griffith University, Gold Coast, QLD Australia

**Keywords:** Healthcare delivery, Cultural and linguistical diversity, Multicultural, Emergency care, Barriers and facilitators, Social ecological model

## Abstract

**Background:**

Worldwide, the culturally and linguistically diverse (CALD) population is increasing, and is predicted to reach 405 million by 2050. The delivery of emergency care for the CALD population can be complex due to cultural, social, and language factors. The extent to which cultural, social, and contextual factors influence care delivery to patients from CALD backgrounds throughout their emergency care journey is unclear. Using a systematic approach, this review aims to map the existing evidence regarding emergency healthcare delivery for patients from CALD backgrounds and uses a social ecological framework to provide a broader perspective on cultural, social, and contextual influence on emergency care delivery.

**Methods:**

The Joanna Briggs Institute (JBI) scoping review methodology will be used to guide this review. The population is patients from CALD backgrounds who received care and emergency care clinicians who provided direct care. The concept is healthcare delivery to patients from CALD backgrounds. The context is emergency care. This review will include quantitative, qualitative, and mixed-methods studies published in English from January 1, 2012, onwards. Searches will be conducted in the databases of CINAHL (EBSCO), MEDLINE (Ovid), Embase (Elsevier), SocINDEX (EBSCO), Scopus (Elsevier), and a web search of Google Scholar. A PRISMA (Preferred Reporting Items for Systematic Reviews and Meta-Analyses) flow diagram will be used to present the search decision process. All included articles will be appraised using the Mixed Methods Appraisal Tool (MMAT). Data will be presented in tabular form and accompanied by a narrative synthesis of the literature.

**Discussion:**

Despite the increased use of emergency care service by patients from CALD backgrounds, there has been no comprehensive review of healthcare delivery to patients from CALD backgrounds in the emergency care context (ED and prehospital settings) that includes consideration of cultural, social, and contextual influences. The results of this scoping review may be used to inform future research and strategies that aim to enhance care delivery and experiences for people from CALD backgrounds who require emergency care.

**Systematic review registration:**

This scoping review has been registered in the Open Science Framework https://doi.org/10.17605/OSF.IO/HTMKQ

**Supplementary Information:**

The online version contains supplementary material available at 10.1186/s13643-024-02579-0.

## Background

Worldwide, the culturally and linguistically diverse (CALD) population reached almost 272 million in 2019, up from 150 million in 2000, and is predicted to reach 405 million by 2050 [1, 2]. About 50% of the CALD population resides in 10 high-income countries, namely Australia, the USA, Canada, several countries in Europe (France, Italy, UK, Germany, Russia), and the middle east (Saudi Arabia, the United Arab Emirates) [1, 3]. Reflecting population growth, emergency service use has also grown [4]. The delivery of effective health services for CALD populations can be complex and challenging for healthcare providers in the prehospital [5, 6] and emergency department (ED) settings [7] due to cultural, social, and language factors which may have an impact on their health practices and the interactions with the healthcare providers in the health system [7, 8].

People from CALD backgrounds comprise around 16–19% of ED presentations [9, 10]. One study has indicated that patients from non-English-speaking backgrounds (NESB) and patients with an English-speaking background but not born in Australia were more likely to visit ED when compared to patients from English-speaking backgrounds but born in Australia because they do not have a general practitioner (GP) [11]. The increased use of emergency services from CALD populations can impact on ED overcrowding, patient safety, culturally unsafe care delivery, and delays in providing diagnostic tests and treatments [9, 12]. The emergency care pathway is multifaceted, with patients presenting to an ED via ambulance, police, self-referral, or referral by a healthcare or community service provider. On arrival at the ED, all patients are triaged to assess their clinical urgency. However, the provision of timely care is reliant on resources within the ED as well as hospital efficiency and capacity to manage patient flow [13]. People’s CALD backgrounds and social expectations not only shape how they regard health and illness but also play a significant role in emergency care decision-making. It is thus essential for hospitals to embrace a culture of quality and patient safety and support the implementation of changes designed to improve care across the healthcare system [14]. This includes opportunities for public health prevention and promotion in the ED through measures such as the display and provision of health promotion materials and advice and guidance to patients [15].

Two recent reviews have been published that focus on access to health services among CALD populations [16] and strategies to improve care for CALD adults in the ED [17]. These reviews are, however, limited to health service access in the Australian context [16] and interventions designed to improve ED performance, outcomes, and experiences (for patients and/or staff) [17]. The extent to which the broader cultural, social, and contextual factors influence on care delivery to patients from CALD backgrounds throughout their emergency care journey is unclear. This review will address these gaps by using a systematic approach to comprehensively map the existing evidence regarding healthcare delivery for patients from CALD backgrounds in emergency care (ED and prehospital settings). The use of a social ecological framework will also provide a broader perspective on cultural, social, and contextual influences on emergency care delivery to patients from CALD backgrounds.

### Aim and review questions

This scoping review aims to provide a comprehensive understanding of existing evidence regarding healthcare delivery to patients from CALD backgrounds in emergency care settings (ED and pre-hospital). Using Joanna Briggs Institute (JBI) population-concept-context (PCC) mnemonic, the main review question guiding this review is as follows: what is the research evidence available with regard to healthcare delivery (concept) for patients from CALD backgrounds and emergency care clinicians providing care (population) in the emergency care settings, including ED and prehospital settings (context)? Sub-questions underpinning this overarching question included the following: (i) how is CALD defined in emergency care research? (ii) what are the demographics, clinical profile, care delivery, and outcomes for patients from CALD backgrounds in emergency care settings? and (iii) what are the facilitators and barriers associated with care delivery in emergency care settings from a broader cultural, social, and contextual perspective?

## Methods

The JBI scoping review methodology will be used to guide this review [18]. The development of the JBI approach has been underpinned by earlier works of Arksey and O’Malley and Levac and colleagues [19, 20]. The JBI scoping review framework entails nine stages of the review process: (i) defining and aligning the objective(s) and question(s); (ii) developing and aligning the inclusion criteria with the objective(s) and question(s); (iii) describing the planned approach to evidence searching, selection, data extraction, and evidence presentation; (iv) searching for the evidence; (v) selecting the evidence; (vi) extracting the evidence; (vii) analyzing the evidence; (viii) presenting the results; and (ix) summarizing the evidence associated with the review purpose, making conclusions and any implication of the findings [18]. We will also consult with an information scientists, key stakeholders, and experts throughout the review process [18] from conception through to dissemination. The team of this scoping review consists of a research librarian (ST), a consumer representative (RL), researchers with experiences in the JBI scoping review process (YLH, JC, CCL, EE), emergency care research (YLH, JC, EE), and social and cultural research (YLH).

The JBI PCC elements will be used to guide the main review question and study inclusion criteria [18]. To understand a broader perspective on cultural, social, and contextual level of influence, a social ecological model (intrapersonal, interpersonal, organizational, community, public policy, physical environment, and cultural levels) [21] will also be used as a framework to map the perceived facilitators and barriers associated with care delivery in emergency care settings to patients from CALD backgrounds. The scoping review has been registered within the Open Science Framework database (registration 10.17605/OSF.IO/HTMKQ). This protocol is being reported in accordance with the Preferred Reporting Items for Systematic Reviews and Meta-Analyses Protocols (PRISMA-P) statement [22] (see checklist in Additional file 1). The scoping review will be reported in accordance with the Preferred Reporting Items for Systematic Reviews and Meta-Analyses extension for Scoping Reviews (PRISMA-ScR) [23]. Definitions of terms used throughout this review protocol are presented in Table 1.

**Table 1:** Definitions of terms

Terms	Definitions
Cultural and linguistic diverse (CALD) population	A CALD population is defined as people from communities with diverse cultural or linguistic affiliations by virtue of country of birth, ancestry, ethnicity, religion, first language spoken/languages spoken at home, or having a parent born overseas [8, 24, 25]
Emergency care (ED and pre-hospital)	An ED is known by various terms such as an accident and emergency department, emergency room, and emergency units [26]. It is defined as a medical treatment facility specializing in emergency medicine that receives, triages, stabilizes, and provides acute care to patients requiring resuscitation, emergency, urgent, semi-urgent, or less-urgent conditions [27]. Prehospital care means “those emergency medical services rendered to the emergency patient for analytic, resuscitative, stabilizing or preventative purposes, precedent to and during transportation of such patient to health care facilities (Page 1)” [28]
Service delivery models/systems	Service delivery models are designed to assist hospitals in meeting the National Emergency Access Targets (NEAT) and to improve the experience of patients’ journeys by providing faster access to safe and quality emergency care [29]

### Inclusion and exclusion criteria

The study eligibility criteria for this review will be guided by the JBI PCC elements [18] and are described in Table 2. Included are studies where the population was focused on people involved with emergency care, there was an explicit focus on healthcare delivery to patients from CALD backgrounds, and the context was the emergency care settings. This review will consider original quantitative (e.g., observational and interventional designs), qualitative (e.g., interview), and mixed-method studies. Studies will be excluded based on the following criteria: not primary research or study, i.e., methodology paper/research protocol, case report, discussion paper; studies with no abstract; studies using primary data published in the format of letters, commentaries, and brief/short communications; and studies with no evidence of ethics approval (or no reason why ethics was not required, i.e., waiver), thesis, editorial, conference abstract, and duplicates. Due to resourcing considerations and limited languages other than English among the team, the search will be limited to peer-reviewed articles that are available in English, published from January 1, 2012, onwards.

**Table 2:** Study eligibility criteria

	**Inclusion criteria**
Population	People involved with emergency care. This includes patients from CALD backgrounds (i.e., ethnicity, immigrants, first language spoken at home) who received care and emergency care clinicians (doctors, nurses, and paramedics) and who provided direct clinical care
Concept	Studies with an explicit focus on healthcare delivery to patients from CALD backgrounds. This includes care provided, outcomes and facilitators, and barriers associated with care delivery
Context	Emergency care, including emergency department and/or prehospital settings

Exclusion criteria include not primary research; studies with no abstract; studies using primary data published in the format of letters, commentaries, and brief/short communications; studies with no evidence of ethics approval, thesis, editorial, conference abstract, and duplicates; and studies which do not meet the inclusion criteria of population, concept, and context

### Information source and search strategy

A three-step search strategy will be used [18]. Electronic databases to be searched for published literature (from January 1, 2012, onwards) will include CINAHL (EBSCO), MEDLINE (Ovid), Embase (Elsevier), SocINDEX (EBSCO), and Scopus (Elsevier). A Google Scholar web search will also be included. The search strategies will be developed and performed in consultation with a research and teaching librarian (ST) and a consumer representative (RL).

In step 1, the initial limited search includes a search of MEDLINE (Ovid) and CINAHL (EBSCO) databases with keywords (i.e., accident and emergency, A&E, prehospital, CALD, multicultural, non-English speaking background) and subject headings (i.e., emergency service, cultural diversity, ethnic groups, healthcare delivery). This initial search is followed by an analysis of the text words contained in the title, abstract, and subject headings of retrieved articles relevant to the topic. The search strategy is refined after consultation with a research and teaching librarian and a consumer representative. In step 2, a second search using the refined search terms are tailored to databases of CINAHL (EBSCO), MEDLINE (Ovid), Embase (Elsevier), SocINDEX (EBSCO), and Scopus (Elsevier). A web search for additional relevant literature using Google Scholar will be performed using search strings. The search strategy for all databases and Google Scholar is presented in Additional file 2. In step 3, the reference list of identified articles will be searched for additional studies.

### Selection of sources of evidence

After the search is completed, all citations will be imported to Covidence [30] for data management and screening. Duplicates will be removed. Two independent researchers (YLH, EE) will screen titles and abstracts. A third researcher (CCL) will moderate where agreement is not achieved. As suggested by JBI [18], pilot testing of source selectors will be performed before the commencement of screening across a team. A random sample of 25 titles and abstracts will be selected and screened by two independent researchers (YLH, EE) and moderated by a third researcher (CCL). The researcher will only start screening when 75% (or greater agreement) is achieved. Full-text articles will be screened by two researchers (YLH, EE). A third researcher (JC) will moderate where agreement is not achieved. Details for the exclusion reasons will be noted in the final report. A PRISMA flow diagram will be used to report details of studies included and excluded at each stage of the study selection process [22].

### Data extraction

Covidence [30] and Microsoft Word software [31] will be used for data extraction. A data charting form will be created in the Microsoft Word software [31] and piloted initially by one researcher (YLH) before applying it within Covidence [30]. Data will be extracted by two researchers (YLH and EE) and crossed-check against original articles by another researcher (JC or CCL) to ensure the validity of extracted data. Potential conflicts will be resolved via discussion with a third researcher (JC), if required. Data extracted from included studies will comprise study author(s), year of publication, country of study, research design and aim, the time frame of the study, study population and sample size, data collection methods, the definition of CALD, and key findings that pertain to the ability to inform this review. This includes the following: (i) demographics, (ii) clinical profile, (iii) care delivery, (iv) outcomes, and (v) barriers and facilitators associated with CALD care delivery in emergency settings. A draft of the data extraction form is provided in Additional file 3.

### Quality appraisal

The Mixed Methods Appraisal Tool (MMAT) [32] will be used to assess the quality of included studies. Two researchers (YLH and EE) will independently assess included articles using the MMAT. If there is any disagreement, three researchers (YLH, EE, and JC) will perform moderation process. The MMAT assessment outcome will be presented in tabular form.

### Data synthesis

The scoping review results will be synthesized and presented in tabular form which will be developed and refined throughout the data extraction. Quantitative, qualitative, and mixed-methods data extracted from included articles will be summarized using numerical counts and percentages. Qualitative data will be thematically sorted into intrapersonal, interpersonal, organizational, community, public policy, physical environment, and cultural levels [21]. The extracted data will be presented as a narrative summary which is aligned with the review aim, review questions, and eligibility criteria (i.e., PCC elements). Specifically, the narrative summary will describe how the results relate to healthcare delivery to patients from CALD backgrounds in emergency care.

## Discussion

This scoping review aims to comprehensively map the existing research evidence regarding healthcare delivery to patients from CALD backgrounds in emergency care. Studies related to care delivery to patients from CALD backgrounds have been reported from several different countries. However, these studies have varying features because of different underlying factors (i.e., health service, population, policy, and guidelines). This review will provide an overview of study characteristics, demographic and clinical profiles, care delivery, and outcomes related to patients from CALD backgrounds who require emergency care. Second, it will articulate the varied definitions of CALD used in the emergency care context. Third, this review will provide a broader perspective on cultural, social, and contextual influences on care delivery to patients from CALD backgrounds. The outcomes of this scoping review will help to inform future research and strategies that aim to enhance care delivery and experiences for people from CALD backgrounds who require emergency care.

### Supplementary Information

Additional file 1: PRISMA-P 2015 Checklist.

Additional file 2. Search strategy for each database. Table 1. CINAHL Search Strategy. Table 2. MEDLINE (Ovid) Search Strategy. Table 3. Embase Search Strategy. Table 4. SocINDEX Search Strategy. Table 5. Scopus (Elsevier) Search Strategy. Table 6. Google Scholar Search Strings.

Additional file 3. Proposed data extraction form.

## Data Availability

Not applicable.

## Abbreviations

CALD: Cultural and linguistic diversity
ED: Emergency department
GP: General practitioner
NESB: Non-English-speaking backgrounds
JBI: Joanna Briggs Institute
MMAT: Mixed Methods Appraisal Tool
PCC: Population, concept, and context
PRISMA-P checklist: Preferred Reporting Items for Systematic Reviews and Meta-Analyses Protocols
PRISMA-ScR: Preferred Reporting Items for Systematic Reviews and Meta-Analyses extension for Scoping Reviews
