# Supplementary material for: Healthcare delivery to patients from culturally and linguistically diverse backgrounds in emergency care: a scoping review protocol
Source: Syst Rev. 2024 Jul 12;13:178. doi: 10.1186/s13643-024-02579-0 (PMC11241862; doi:10.1186/s13643-024-02579-0)
Supplement: Supplementary file 2 — Additional file 2. Search strategy for each database. Table 1. CINAHL Search Strategy. Table 2. MEDLINE (Ovid) Search Strategy. Table 3. Embase Search Strategy. Table 4. SocINDEX Search Strategy. Table 5. Scopus (Elsevier) Search Strategy. Table 6. Google Scholar Search Strings. [file 13643_2024_2579_MOESM2_ESM.docx]

**Additional file 2. Search strategy for each database**

**Table 1. CINAHL Search Strategy**

| **Search ID** | **Search Terms: CINAHL (Ebsco)** |
| --- | --- |
| S1 | (MH "Emergency Medical Services") OR (MH "Emergency Service") |
| S2 | TI ("accident and emergency" or "A&E" or "ED" or "A and E" or “pre-hospital”) OR AB ("accident and emergency" or "A&E" or "ED" or "A and E" or pre-hospital) |
| S3 | TI ((emergency N2 (unit or units or room or rooms or ward or wards or department* or service*)) OR AB ((emergency N2 (unit or units or room or rooms or ward or wards or department* or service*)) |
| S4 | S1 OR S2 OR S3 |
| S5 | (MH "Cultural Diversity") OR (MH "Cultural Safety") OR (MH "Cultural Values") OR (MH “Ethnic Groups”) OR (MH “Immigrants”) OR (“Language”) |
| S6 | (MH "Minority Groups") |
| S7 | (MH "Refugees") |
| S8 | TI (CALD or "cultural and linguistic diversity") OR AB (CALD or "cultural and linguistic diversity") |
| S9 | TI ((cultur* or liguistic* or language or racial* or ethnic*) N2 (divers* or barrier*)) OR AB ((cultur* or liguistic* or language or racial* or ethnic*) N2 (divers* or barrier*)) |
| S10 | TI ((ethnic or racial or linguistic or language) N2 minorit*) OR AB ((ethnic or racial or linguistic or language) N2 minorit*) |
| S11 | TI (cultural N2 (awareness or competence)) OR AB (cultural N2 (awareness or competence)) |
| S12 | TI (non-english or non-white or non-western non-australian*) OR AB (non-english or non-white or non-western non-australian*) |
| S13 | TI (migrant* or immigrant* or foreign-born or refugee* or (born N1 overseas)) OR AB (migrant* or immigrant* or foreign-born or refugee* or (born N1 overseas) ) |
| S14 | TI (nesb or "non english speaking" or LOTE) OR AB (nesb or "non english speaking" or LOTE) |
| S15 | TI ((language N1 "other than english") or "english as a second laguage") OR AB ((language N1 "other than english") or "english as a second laguage") |
| S16 | TI (english N1 (limited or lacking or lack or lacks)) OR AB (english N1 (limited or lacking or lack or lacks)) |
| S17 | TI (multicultural or multi-cultural) OR AB (multicultural or multi-cultural) |
| S18 | TI multi-ethnic* OR AB multi-ethnic* |
| S19 | S5 OR S6 OR S7 OR S8 OR S9 OR S10 OR S11 OR S12 OR S13 OR S14 OR S15 OR S16 OR S17 OR S18 |
| S20 | S4 AND S19 |
| S21 | (MH "Health Care Delivery") OR (MH "Health Care Delivery, Integrated") |
| S22 | TI ((model* N2 (service* or care)) OR AB ((model* N2 (service* or care)) |
| S23 | TI ((service* N2 (framework* or infrastructure)) OR AB ((service* N2 (framework* or infrastructure)) |
| S24 | TI ((service* N2 (initiativ* or configurat* or deliver* or capabilit*)) OR AB ((service* N2 (initiativ* or configurat* or deliver* or capabilit*)) |
| S25 | TI ((intervention* N2 (target* or service* or strateg*)) OR AB ((intervention* N2 (target* or service* or strateg*)) |
| S26 | (MH "Patient Care") OR (MH "Patient Centered Care") OR (MH "Patient Navigation") OR (MH "Family Centered Care") OR (MH "Case Management") |
| S27 | (MH "Patient Care Plans") |
| S28 | (MH "Critical Path") |
| S29 | TI ("patient care" N2 (management or team or plan*)) OR AB ("patient care" N2 (management or team or plan*)) |
| S30 | TI ((patient or critical) N2 pathway*) OR AB ((patient or critical) N2 pathway*) |
| S31 | TI ((delivery or model*) N2 (care or healthcare or health-care)) OR AB ((delivery or model*) N2 (care or healthcare or health-care) ) |
| S32 | (MH "Organizational Culture") OR (MH "Program Development") OR (MH "Quality Management, Organizational") |
| S33 | (MH "Health Services Accessibility") |
| S34 | TI barrier* N5 facilitator* OR AB barrier* N5 facilitator* |
| S35 | S21 OR S22 OR S23 OR S24 OR S25 OR S26 OR S27 OR S28 OR S29 OR S30 OR S31 OR S32 OR S33 OR S34 |
| S36 | S20 AND S35  **Limiters** - Published Date: 20120101-current; English Language |

**Table 2. MEDLINE (Ovid) Search Strategy**

| **Search ID** | **Search Terms: Medline (Ovid)** |
| --- | --- |
| 1 | cultural diversity/ |
| 2 | population groups/ or racial groups/ or ethnicity/ or refugees/ or "transients and migrants"/ |
| 3 | "ethnic and racial minorities"/ or minority groups/ |
| 4 | "Emigrants and Immigrants"/ |
| 5 | Language/ |
| 6 | (CALD or "cultural and linguistic diversity").tw. |
| 7 | (cultural* adj2 (aware* or competence)).tw. |
| 8 | ((cultur* or liguistic* or language or racial* or ethnic*) adj2 (divers* or barrier*)).tw. |
| 9 | ((ethnic or racial or linguistic or language) adj2 minorit*).tw. |
| 10 | (non-english or non-white or non-western).tw. |
| 11 | non-australian*.tw. |
| 12 | (migrant* or immigrant* or foreign-born or refugee* or (born adj2 overseas)).tw. |
| 13 | (english adj3 language adj3 (second or "other than")).tw. |
| 14 | (nesb or "non english speaking" or LOTE).tw. |
| 15 | (english adj2 (limited or lack or lacking)).tw. |
| 16 | (multicultural or multi-cultural).tw. |
| 17 | multi-ethnic*.tw. |
| 18 | or/1-17 |
| 19 | Emergency Service, Hospital/ |
| 20 | Emergency Medical Services/ |
| 21 | (emergency adj2 (unit or units or room or rooms or ward or wards or department* or service*)).tw. |
| 22 | ("accident and emergency" or "A&E" or "ED" or "A and E" or pre-hospital).tw. |
| 23 | or/19-22 |
| 24 | 18 and 23 |
| 25 | patient care management/ or "delivery of health care"/ or disease management/ or patient care team/ or "quality of health care"/ |
| 26 | patient care planning/ or case management/ or critical pathways/ or patient-centered care/ or progressive patient care/ |
| 27 | (patient care adj2 (management or team or plan*)).tw. |
| 28 | ((patient or critical) adj2 pathway*).tw. |
| 29 | ((delivery or model?) adj2 (care or healthcare or health-care)).tw. |
| 30 | (service adj2 (framework* or infrastructure)).tw. |
| 31 | (service* adj2 (initiativ* or configurat* or deliver* or capabilit*)).tw. |
| 32 | Patient-Centered Care/ |
| 33 | "organization and administration"/ or organizational culture/ or organizational innovation/ or organizational objectives/ |
| 34 | og.fs. |
| 35 | (barrier* adj5 facilitator*).tw. |
| 36 | or/25-35 |
| 37 | 24 and 36 |
| 38 | limit 37 to (english language and yr="2012 -Current") |

**Table 3. Embase Search Strategy**

| **Search Terms: Embase** |
| --- |
| #34 #31 NOT #32 AND [embase]/lim |
| #33 #31 NOT #32 |
| #32 #31 AND 'Conference Abstract'/it |
| #31 #20 AND #29 AND [english]/lim |
| #30 #20 AND #29 |
| #29 #21 OR #22 OR #23 OR #24 OR #25 OR #26 OR #27 OR #28 |
| #28 (barrier* NEAR/4 facilitator*):ti,ab |
| #27 'organizational culture'/de OR 'organization and management'/de |
| #26 (service* NEAR/1 (initiativ* OR configurat* OR deliver* OR capabilit*)):ti,ab |
| #25 (service NEAR/2 (framework* OR infrastructure)):ti,ab |
| #24 ((delivery OR model*) NEAR/1 (care OR healthcare OR 'health care')):ti,ab |
| #23 ((patient OR critical) NEAR/1 pathway*):ti,ab |
| #22 ('patient care' NEAR/1 (management OR team OR plan*)):ti,ab |
| #21 'patient care'/de OR 'case management'/de OR 'collaborative care team'/de OR 'patient care planning'/de OR 'health care delivery'/de OR 'clinical pathway'/de OR 'health care quality'/de |
| #20 #15 AND #19 |
| #19 #16 OR #17 OR #18 |
| #18 'accident and emergency':ti,ab OR 'a&e':ti,ab OR 'ed':ti,ab OR 'a and e':ti,ab OR 'pre hospital':ti,ab |
| #17 (emergency NEAR/1 (unit OR units OR room OR rooms OR ward OR wards OR department* OR service*)):ti,ab |
| #16 'hospital emergency service'/de OR 'emergency health service'/de |
| #15 #1 OR #2 OR #3 OR #4 OR #5 OR #6 OR #7 OR #8 OR #9 OR #10 OR #11 OR #12 OR #13 OR #14 |
| #14 'multi ethnic*':ti,ab |
| #13 multicultural:ti,ab OR 'multi cultural':ti,ab |
| #12 (english NEAR/1 (limited OR lack OR lacking)):ti,ab |
| #11 nesb:ti,ab OR 'non english speaking':ti,ab OR lote:ti,ab |
| #10 (english NEAR/2 language NEAR/2 (second OR 'other than')):ti,ab |
| #9 migrant*:ti,ab OR immigrant*:ti,ab OR 'foreign born':ti,ab OR refugee*:ti,ab OR (born NEAR/1 overseas):ti,ab |
| #8 'non australian':ti,ab OR 'non english':ti,ab OR 'non white':ti,ab OR 'non western':ti,ab |
| #7 ((ethnic OR racial OR linguistic OR language) NEAR/1 minorit*):ti,ab |
| #6 ((cultur* OR liguistic* OR language OR racial* OR ethnic*) NEAR/1 (divers* OR barrier*)):ti,ab |
| #5 (cultural* NEAR/1 (aware* OR competence)):ti,ab |
| #4 cald:ti,ab OR 'cultural and linguistic diversity':ti,ab |
| #3 'minority group'/de |
| #2 'ethnicity'/de OR 'ethnic or racial aspects'/de OR 'cultural factor'/de OR 'ethnic difference'/de OR 'race'/de OR 'race difference'/exp OR 'immigrant'/de |
| #1 'cultural diversity'/de |

**Table 4. SocINDEX Search Strategy**

| S1 | DE "EMERGENCY medical services" OR DE "EMERGENCY medicine" OR DE "PARAMEDICINE" |
| --- | --- |
| S2 | TI ("accident and emergency" or "A&E" or "ED" or "A and E" or “pre-hospital”) OR AB ("accident and emergency" or "A&E" or "ED" or "A and E" or pre-hospital) |
| S3 | TI ((emergency N2 (unit or units or room or rooms or ward or wards or department* or service*)) OR AB ((emergency N2 (unit or units or room or rooms or ward or wards or department* or service*)) |
| S4 | S1 OR S2 OR S3 |
| S5 | DE "MULTICULTURALISM" OR DE "BICULTURALISM" OR DE "CULTURAL pluralism" OR DE "MINORITIES" OR DE "MULTILINGUALISM" OR DE "PEOPLE of color" OR DE "PLURAL societies" |
| S6 | DE "CULTURAL literacy" OR DE "CULTURAL awareness" OR DE "CULTURAL values" OR DE "CULTURAL identity" |
| S7 | DE "ETHNIC groups" OR DE "ETHNOLINGUISTIC groups" OR DE "MULTIRACIAL people" OR DE "ETHNIC differences" OR DE "ETHNICITY" |
| S8 | DE "IMMIGRANTS" OR DE "IMMIGRANT children" OR DE "IMMIGRANT men" OR DE "OLDER immigrants" OR DE "WOMEN immigrants" OR DE "REFUGEES" |
| S9 | DE "CULTURAL competence" OR DE "CROSS-cultural communication" |
| S10 | DE "CULTURAL prejudices" OR DE "CULTURE conflict" |
| S11 | DE "CULTURAL intelligence" |
| S12 | TI (CALD or "cultural and linguistic diversity") OR AB (CALD or "cultural and linguistic diversity") |
| S13 | TI ((cultur* or liguistic* or language or racial* or ethnic*) N2 (divers* or barrier*)) OR AB ((cultur* or liguistic* or language or racial* or ethnic*) N2 (divers* or barrier*)) |
| S14 | TI (cultural N2 (awareness or competence)) OR AB (cultural N2 (awareness or competence)) |
| S15 | TI (non-english or non-white or non-western non-australian*) OR AB (non-english or non-white or non-western non-australian*) |
| S16 | TI (migrant* or immigrant* or foreign-born or refugee* or (born N1 overseas)) OR AB (migrant* or immigrant* or foreign-born or refugee* or (born N1 overseas)) |
| S17 | TI ((language N1 "other than english") or "english as a second laguage") OR AB ((language N1 "other than english") or "english as a second laguage") |
| S18 | TI (english N1 (limited or lacking or lack or lacks)) OR AB (english N1 (limited or lacking or lack or lacks)) |
| S19 | TI (multicultural or multi-cultural) OR AB (multicultural or multi-cultural) |
| S20 | TI multi-ethnic* OR AB multi-ethnic* |
| S21 | S5 OR S6 OR S7 OR S8 OR S9 OR S10 OR S11 OR S12 OR S13 OR S14 OR S15 OR S16 OR S17 OR S18 OR S19 OR S20 |
| S22 | S4 AND S21 |
| S23 | DE "MEDICAL care" OR DE "HEALTH services accessibility" OR DE "HOSPITAL care" OR DE "PATIENT-centered care" OR DE "TRANSCULTURAL medical care" OR DE "MEDICINE & culture" OR DE "PATIENT care" OR DE "PATIENT safety" |
| S24 | TI ((model* N2 (service* or care)) OR AB ((model* N2 (service* or care)) |
| S25 | TI ((service* N2 (framework* or infrastructure)) OR AB ((service* N2 (framework* or infrastructure)) |
| S26 | TI ((service* N2 (initiativ* or configurat* or deliver* or capabilit*)) OR AB ((service* N2 (initiativ* or configurat* or deliver* or capabilit*)) |
| S27 | TI ((intervention* N2 (target* or service* or strateg*)) OR AB ((intervention* N2 (target* or service* or strateg*)) |
| S28 | TI ("patient care" N2 (management or team or plan*)) OR AB ("patient care" N2 (management or team or plan*)) |
| S29 | TI ((patient or critical) N2 pathway*) OR AB ((patient or critical) N2 pathway*) |
| S30 | TI ((delivery or model*) N2 (care or healthcare or health-care)) OR AB ((delivery or model*) N2 (care or healthcare or health-care)) |
| S31 | DE "ORGANIZATIONAL behavior" OR DE "MEDICAL quality control" OR DE "COMMUNICATION barriers" OR DE "ETHNIC barriers" |
| S32 | TI barrier* N5 facilitator* OR AB barrier* N5 facilitator* |
| S33 | S23 OR S24 OR S25 OR S26 OR S27 OR S28 OR S29 OR S30 OR S31 OR S32 |
| S34 | S22 AND S33 |
| S35 | S22 AND S33  Limiters - Date of Publication: 20120101-current; Language: English |

**Table 5. Scopus (Elsevier) Search Strategy**

| TITLE-ABS-KEY (*cald*  OR  *"cultural and linguistic diversity"*  OR  *non-english*  OR  *non-white*  OR  *non-western*  OR  *non-australian**  OR  *migrant**  OR  *immigrant**  OR  *foreign-born*  OR  *refugee**  OR  *nesb*   OR  *"non english speaking"*  OR  *lote*  AND *multicultural*  OR  *multi-cultural*  OR  *multi-ethnic** )   OR  TITLE-ABS-KEY ( *cultural**  W/1  ( *aware**  OR  *competence* ))   OR TITLE-ABS-KEY ((*cultur**  OR  *liguistic**  OR  *language*  OR  *racial**  OR  *ethnic**) W/1 (*divers**  OR  *barrier**))   OR TITLE-ABS-KEY ( ( *ethnic*  OR  *racial*  OR  *linguistic*  OR  *language* )  W/1  *minorit** )   OR  TITLE-ABS-KEY ( *born*  W/1  *overseas* )   OR  TITLE-ABS-KEY ( *english*  W/1  ( *limited*  OR  *lack*  OR  *lacking* ))   OR  TITLE-ABS-KEY ( *english*  W/2  *language*  AND *w//2*  ( *second*  OR  *"other than"* ))   AND   TITLE-ABS-KEY (*emergency*  W/2  (*unit*  OR  *units*  OR  *room*  OR  *rooms*  OR  *ward*  OR  *wards*  OR  *department**   OR  *service**)) OR  TITLE-ABS-KEY (*"accident and emergency"*  OR  *"A&E"*  OR  *"ED"*  OR  *"A and E"*  OR  *pre-hospital* )  AND   ((TITLE-ABS-KEY ( *barrier**  W/5  *facilitator**)   OR  TITLE-ABS-KEY ( *"patient care"*  W/1  ( *management*  OR  *team*  OR  *plan**  OR  *delivery* ))   OR  TITLE-ABS-KEY ( ( *patient*  OR  *critical* )  W/1  *path** )   OR  TITLE-ABS-KEY ( ( *delivery*  OR  *model** )  W/1  ( *care*  OR  *healthcare*  OR  *health-care* ))   OR  TITLE-ABS-KEY ( *service*  W/1  ( *framework**  OR  *infrastructure*  OR  *initiativ**  OR  *configurat**  OR  *deliver**   OR  *capabilit** ))   AND  ( LIMIT-TO ( LANGUAGE ,  *"English"* )) |
| --- |

**Table 6. Google Scholar Search Strings**

| **#** | **Search strings** |
| --- | --- |
| 1 | CALD and care delivery and emergency care |
| 2 | (CALD OR "culturally and linguistically diverse" OR ethnicity OR immigrants) AND (health care delivery OR intervention OR facilitator OR barrier) AND emergency |
