# Supplementary material for: Healthcare delivery to patients from culturally and linguistically diverse backgrounds in emergency care: a scoping review protocol
Source: Syst Rev. 2024 Jul 12;13:178. doi: 10.1186/s13643-024-02579-0 (PMC11241862; doi:10.1186/s13643-024-02579-0)
Supplement: Supplementary file 3 — Additional file 3. Proposed data extraction form. [file 13643_2024_2579_MOESM3_ESM.docx]

**Additional file 3. Proposed data extraction form**

| **Reviewer:** | |
| --- | --- |
| **Date** | |
| **Key elements** | **Reviewer’s response** |
| Author(s), Year, Country |  |
| Research Design |  |
| Aim/Purpose |  |
| Study population and sample size |  |
| Time frame of study |  |
| Data collection/Recruitment procedure |  |
| Definition of CALD |  |
| Demographic profile (i.e., age, gender, country of birth and language spoken) |  |
| Clinical profile (i.e., model of arrival, reasons for presentation, time of day, triage category and ICD-10-CM diagnosis code) |  |
| Care delivery (i.e., referrals, consultations, interpreter service use, follow up and diagnostic tests) |  |
| Outcomes (i.e., time of the first meaning full treatments,  waiting time to be seen by a doctor, ED length of stay and hospital length of stay) |  |
| Barriers and facilitators associated with CALD care delivery in emergency settings (data will be thematically sorted into intrapersonal, interpersonal, organizational, community, public policy, physical environment, and cultural levels) |  |
